# Anatomical Variation in Quadriceps Angle With Regard to Different Anthropometric Parameters in a Tertiary Care Center in Northern India: A Descriptive Study

**DOI:** 10.7759/cureus.34224

**Published:** 2023-01-26

**Authors:** Kumar Shantanu, Shailendra Singh, Deepak Kumar, Arpit Singh, Prakash G Tewari, Pranjal Gupta

**Affiliations:** 1 Department of Orthopaedic Surgery, King George's Medical University, Lucknow, IND

**Keywords:** age, bmi, weight, height, bilateral variation, femur length, femoral bicondylar distance, quadriceps angle, q-angle

## Abstract

Background: The quadriceps angle, commonly known as the Q-angle, is the angle formed between the vectors of the pull of the quadriceps muscle and the patellar tendon. The literature varies in terms of the values of Q angles measured by various researchers. It is well appreciated that the normal Q-angle should fall between 12° and 20°, with males being at the lower end of this range and females having higher measurements. An increase in Q-angle beyond the normal range has been associated with knee extensor dysfunction leading to patellar instability. Keeping in mind the clinical and biomechanical importance of the Q-angle, the aim of this study was to compare and establish the range of the Q-angle in healthy individuals and evaluate its variations with respect to age, weight, height, gender, dominant side, and femoral bicondylar distance. These observations will be helpful for sports therapists in understanding the evaluation of Q-angle in athletes as a prognostic value for probable knee pathologies that may appear in the future.

Methods: The current study was conducted at a tertiary care center, and a total of 100 healthy adults between the ages of 18 and 35 were enrolled in the study (50 males and 50 females), following which their Q-angles, bicondylar distances, and femur lengths were measured. Individuals with any lower limb injury that resulted in a ligamentous, muscular, or bony defect; any spinal or neurological injury; any diagnosed knee disorder, such as a fracture, acute or chronic knee pain, patellar dislocation, or prior orthopaedic surgery in the lower extremities, were excluded from the study. Data were analyzed using paired t-tests, independent sample t-tests, ANOVA, and Pearson correlation coefficients.

Result and conclusion: The mean Q-angle in males was found to be 11.14° ± 1.9° on the right side and 10.84° ± 1.86° on the left side. In females, it was found to be 13.68° ± 1.87° on the right side and 13.61° ± 2.04° on the left side. Among males, right and left Q-angles showed significant positive correlations with height, weight, BMI, right femur length, left femur length, right bicondylar distance, and left bicondylar distance (p<0.05). The highest correlation was found between weight and BMI. Among females, the right Q-angle showed significant positive correlations with weight and BMI (p<0.05). The highest correlation was found with weight.

## Introduction

In 1964, Brattstorm originally defined the quadriceps angle as being an acute angle formed between the patellar tendon and quadriceps muscles [1]. The Q-angle can be measured in a supine or standing position. Measurements taken in the standing position are usually more suitable due to the normal weight-bearing forces applied to the knee joint during daily activity [2]. The Q-angle is formed between a line representing the resultant line of force of the quadriceps, formed by joining a point at the anterior superior iliac spine (ASIS) to the centre of the patella, and another line joining the centre of the patella to a point on the tibial tuberosity. Various researchers have described different values of the Q-angle. The normal values of the Q-angle usually fall between a range of 12° and 20°; the males mostly have a lower value, whereas the females tend to have higher values [3-5]. In his study, Bohannon did not describe a range for normal values, but he considered Q-angles >20˚ as excessive [6]. The value of the Q-angle is usually considered excessive when the increase in the lateral pull by the quadriceps on the patella results in an increased chance of patello-femoral disorders [7].

The Q-angle has come to be acknowledged as a crucial component in evaluating knee biomechanics. Q-angle has been identified as a risk factor for ligamentous injuries of the knee in female athletes [8].

An excessive Q-angle implies an increase in the biomechanical stresses at the knee joint during movements [8] as it interferes with the normal patellar tracking in the trochlear groove [9-10]. Over the course of time, notably with sports activities, it causes the wearing away of cartilage on the undersurface of the patella, resulting in loosening and damage to the articular surface of the knee [11]. Moreover, an abnormally increased Q-angle leads to increased foot pronation, which in turn causes an increase in tibial internal rotation, resulting in a change in the quadriceps mechanism and lateral tracking of the patella [12]. Eventually, this results in an accelerated progression from knee dysfunction to patello-femoral arthralgia, finally leading to degenerative joint disease. Correcting foot pronation can often decrease the harmful effects of an abnormal Q-angle [13].

An increase in Q-angle beyond the normal range is looked at as indicative of extensor mechanism misalignment and has been associated with knee joint hypermobility, patello-femoral pain syndrome, chondromalacia patellae, patella alta and patellar instability with recurrent subluxation of the patella [14-17]. Moreover, its role in determining other lower extremity sports injuries has been documented [6].

Studies have reported that the Q-angle is found to be greater in individuals suffering from pathological conditions of the patello-femoral joint [17-24] and varies with gender and anthropometric variables [25-28].

Owing to its clinical and biomechanical importance, this study was conducted to compare and establish the range of the Q-angle in healthy individuals and evaluate its variations with respect to age, weight, height, gender, dominant side, and femoral intercondylar distance. The observations made will be helpful for sports therapists in understanding the evaluation of Q-angle in athletes as a prognostic tool for probable knee pathologies that may appear in the future. The observations will also be useful to physiotherapists and orthopaedic surgeons for a better understanding of patello-femoral disorders.

## Materials and methods

The study was a cross-sectional study done over a period of one year, from September 2020 to August 2021. The study included 100 volunteers (50 males and 50 females), without any injury to the lower limb that led to a ligamentous, muscular, or bony defect; any spinal or neurological injury; any diagnosed knee disorder; acute or chronic knee pain; dislocation of the patella; or prior orthopaedic surgery of the lower extremities. The sample size was calculated on the basis of the variation in Q-angle and its mean using the formula:

$n= {(Z\alpha + Z\beta )^2\sigma ^2}/d^2$

where s = 2.2, the max SD of Q-angle; d = 0.1 times mean Q-angle (=8.6); type I error α = 5% corresponding to 95% confidence level; type II error β = 10% for detecting results with 90% power of study; data loss = 10%. So the minimum required sample size n = 95.

The volunteers were taken from amongst the people attending the outpatient Department of Orthopaedic Surgery at King George’s Medical University. All the individuals were between the ages of 18 and 35. Clearance was given by the institutional ethics committee (104th ECM 11 B-Thesis/P47). The procedure was explained to the subjects, and written consent was taken. The patient’s age, gender, weight, height, and dominant side were noted on a specific investigation sheet. A single investigator took the measurements to minimize observer bias. The measurements were done bilaterally in all subjects, in standing positions.

**Figure 1:**
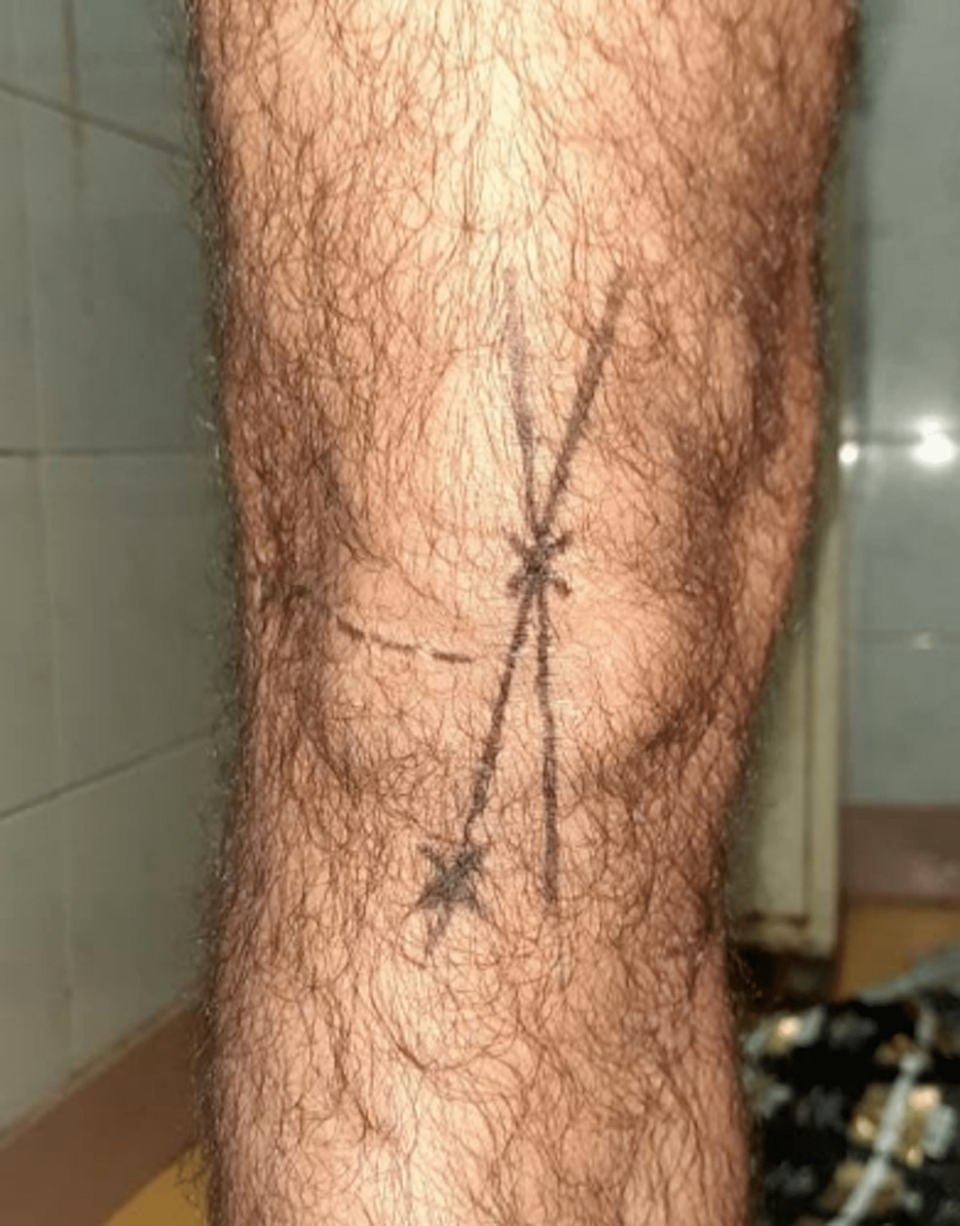
Bony landmarks used to construct the lines for measurement of the Q-angle

For measurement of the Q-angle, using a marker pen, the anterior superior iliac spine, the centre of the patella, and the centre of the tibial tuberosity were marked. The outline of the patella was drawn by palpating its borders. The point where the maximum vertical and transverse diameters of the patella intersect was marked as the centre of the patella. The centre of the tibial tuberosity was marked as the point of maximum prominence of the tibial tuberosity. Two lines were drawn as below: (a) from the anterior superior iliac spine to the centre of the patella, and (b) from the centre of the patella to the centre of the tibial tuberosity, as shown in Figure 1. The fulcrum of the goniometer was placed at the centre of the patella. One arm was directed towards the tibial tuberosity. The other arm was directed towards the anterior superior iliac spine. The Q-angle in degrees was thus measured on both sides [23].

**Figure 2:**
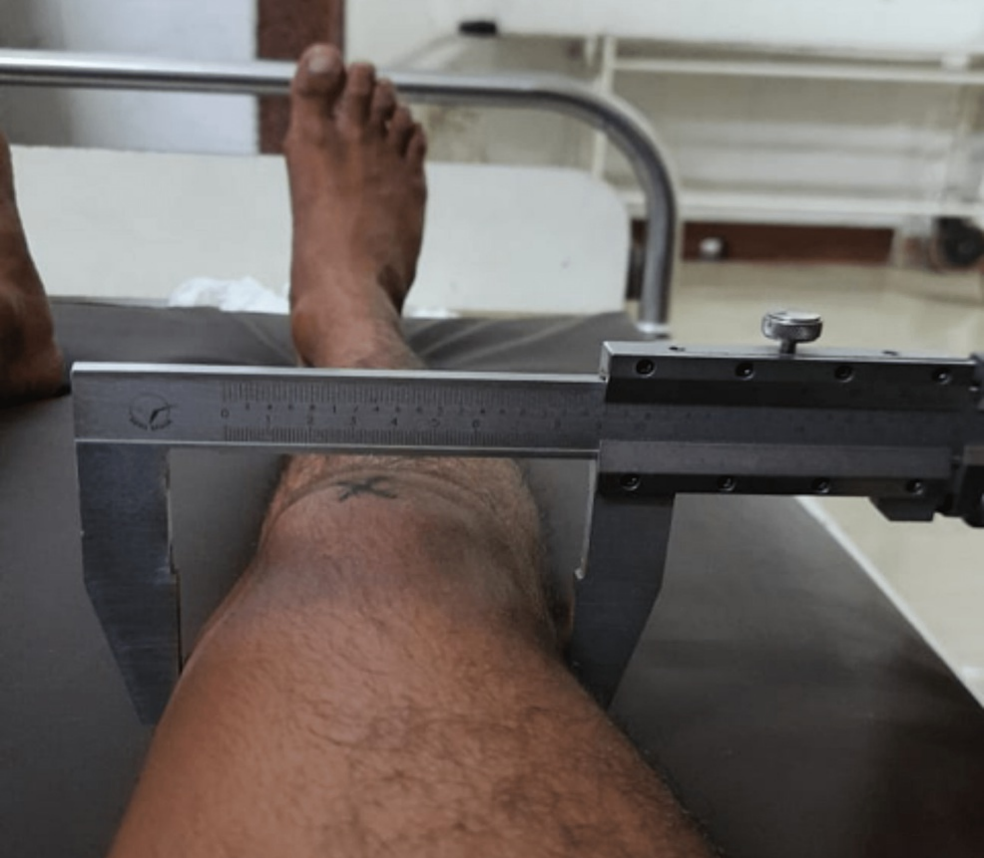
Use of manual caliper to measure the distal femoral intercondylar distance

A manual caliper, scaled from 0 cm to 60 cm and with a marginal error of ±0.1 mm, was used to measure the intercondylar distance of the distal femur bilaterally in each volunteer. The subject was first made to lie supine in the anatomical position, and then the leg was made to flex to 90˚, making the femoral condyles prominent and easily palpable. The fixed arm of the calliper was placed on the lateral condyle, and the movable arm was placed on the medial condyle; the condylar distances were noted for each side on the participant’s investigation sheet (Figure 2).

**Figure 3:**
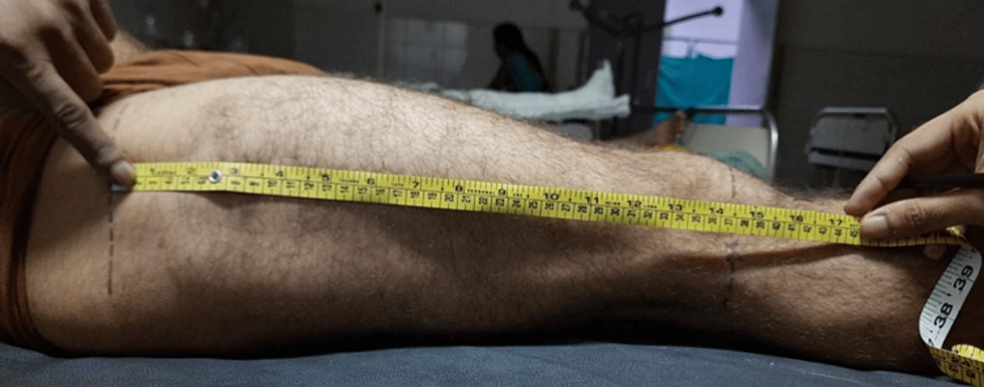
Use of a measuring tape to measure the femur length

A stretchable measuring tape was used to measure the femur length. The subject was first made to lie supine. The length is measured from the greater trochanter to the lateral condyle (Figure 3).

## Results

The patients in the study were divided into two groups on the basis of age (<25 years and ≥25 years), body mass index (<25 kg/m^2^ and 25-30 kg/m^2^), and dominant side (left and right). The mean height of males in the study was 164 ± 9.59 cm, whereas that of females was found to be 157.08 ± 6.46 cm, thus showing a significant difference between the heights of males and females, with males having a higher average height. The mean weight of males in the study was 60.17 ± 12.09 kg, whereas that of females was found to be 56.48 ± 7.34 kg. No significant difference was found in the weights of males and females (p=0.068).

The average right-side femur length in males was found to be 40.32 ± 3.37 cm on the right side and 40.26 ± 3.1 cm on the left side. In females, it was found to be 39.20 ± 2.12 cm on the right side and 39.14 ± 2.07 cm on the left side. Hence, the mean femur length of males was relatively greater than that of females. However, no significant differences in femur lengths were detected on either the left or right sides. The average right-sided bicondylar distance of males was found to be 8.87 ± 1.01 cm and 8.8 ± 1.1 cm on the left side. In females, it was found to be 8.45 ± 0.76 cm on the right side and 8.49 ± 0.72 cm on the left side.

**Figure 4:**
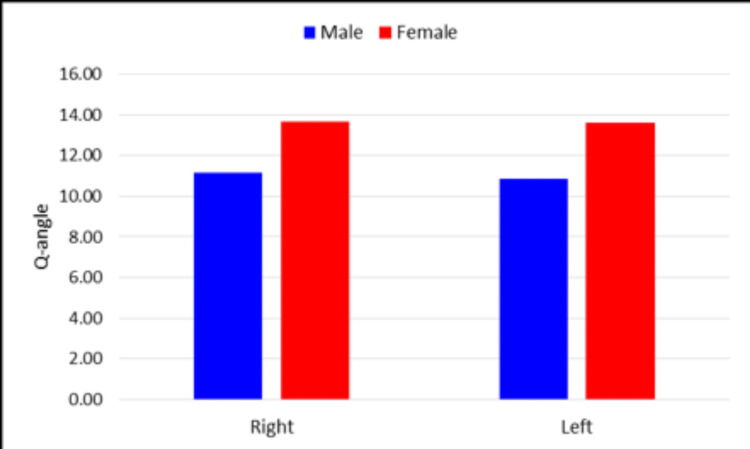
Mean Q-angle values in males and females

The mean Q-angle in our study in males was found to be 11.14° ± 1.9° on the right side and 10.84° ± 1.86° on the left side. In females, it was found to be 13.68° ± 1.87° on the right side and 13.61° ± 2.04° on the left side. There was a significant difference between male and female Q-angles on both the left and right sides (Figure 4).

On comparing the values of Q-angles with the dominant side, we found no significant differences. Among the males, the left side was found to be dominant in four individuals, whereas 46 males had a dominant right side. In the case of females, the left side was dominant in five individuals, whereas the right side was dominant in 45 individuals.

**Figure 5:**
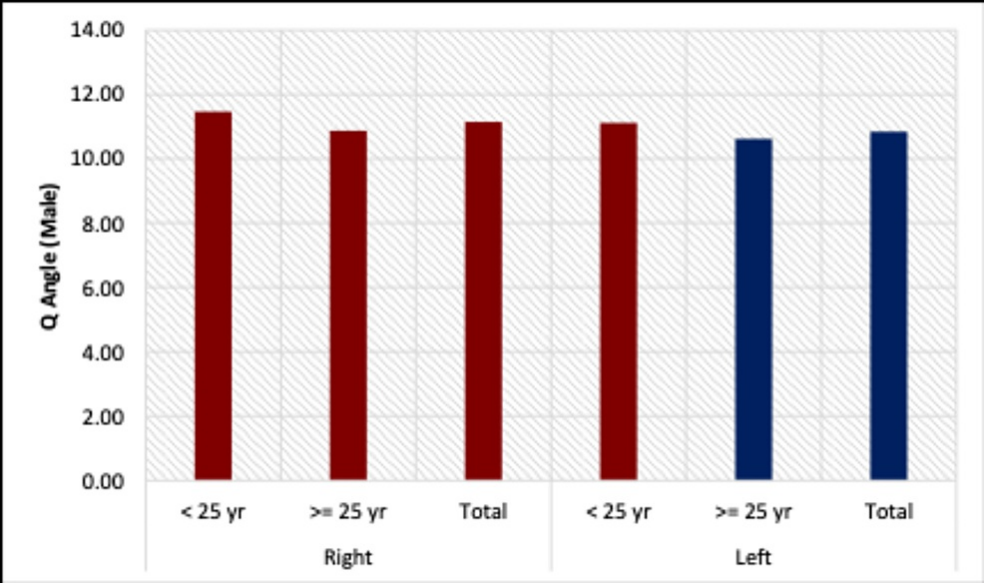
Comparison of Q-angles in age groups in males on both sides

**Figure 6:**
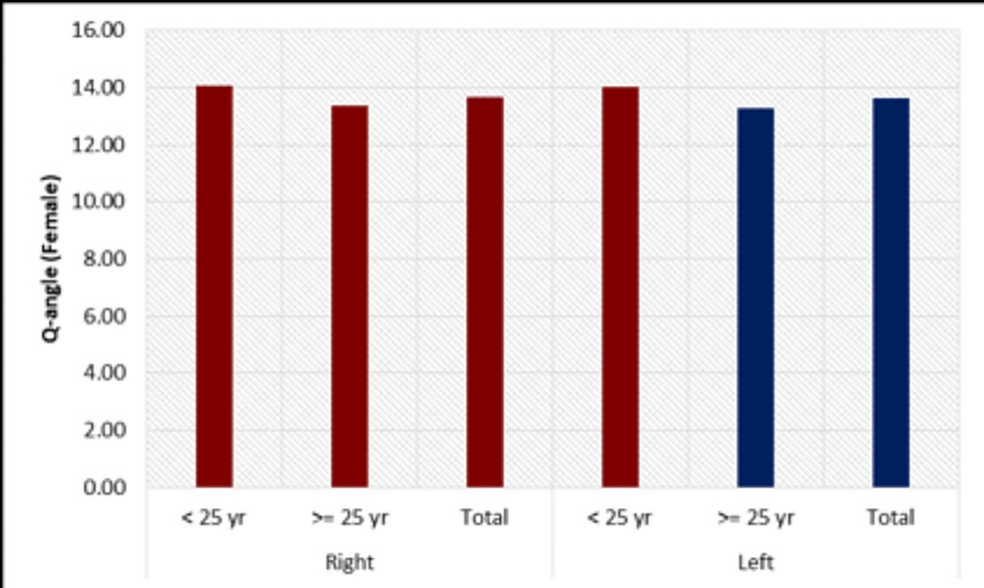
Comparison of Q-angles in age groups, in females on both sides

On comparing the bicondylar distances, femur lengths, and Q-angles with various age groups in males, it was found that both the average right and left side femur lengths, bicondylar distances, and Q-angles of males for the ≥25-year age group were greater than that of <25 year age group. However, it was not significantly different (Figure 5). In females, the bicondylar distance, femur length, and Q-angle on both left and right sides were greater for the less than 25-year-old age group, but these differences were not statistically significant (Figure 6).

**Figure 7:**
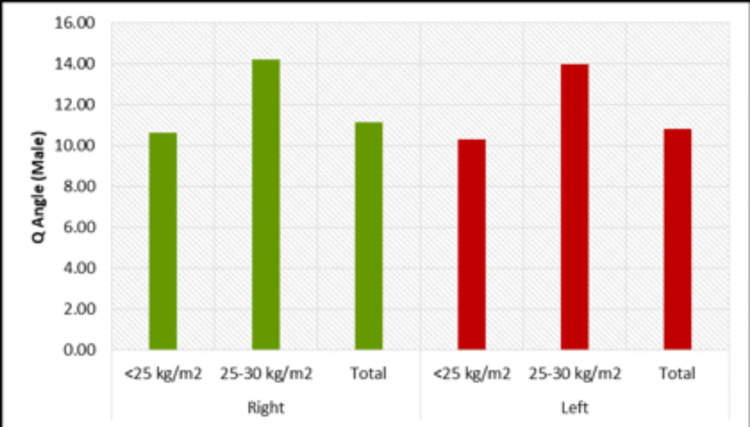
Variation of Q angles as per BMI on both sides in males

**Figure 8:**
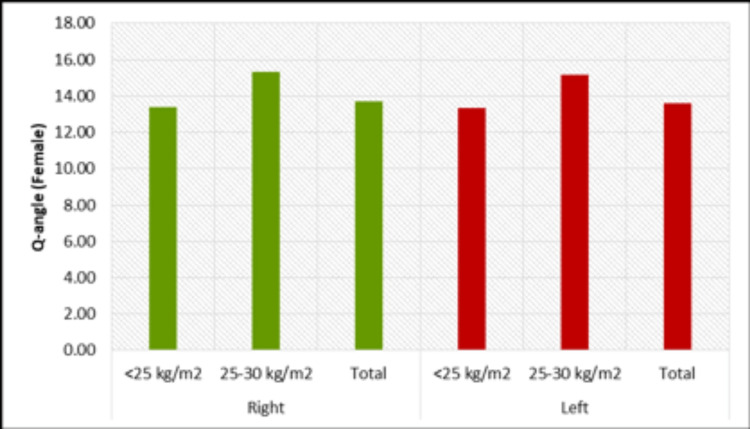
Variation of Q angle with BMI on both sides, in females

In the study, we divided the volunteers into two groups based on their BMI and compared their femur length and bicondylar distance. In males, the mean bicondylar distance and Q-angle on both left and right sides were greater in the BMI 25-30 kg/m^2^ group than in those with a BMI less than 25 kg/m^2^ and the difference was found to be significant. The mean femur length was also found to be higher in the BMI 25-30 kg/m^2^ group than in the BMI less than 25 kg/m^2^ group, although the difference was not statistically significant. In females, the mean femur length on both the left and right sides was greater in the BMI less than 25 kg/m^2^ group than in the BMI 25-30 kg/m^2^ group, but the difference was not found to be significant. On both the left and right sides, the mean bicondylar distance of female volunteers was found to be higher in the BMI 25-30 kg/m^2^ group than in the BMI less than 25 kg/m^2^ group. This difference was statistically significant on the left side but on the right side was not statistically significant. Comparing the Q-angle with BMI groups found that the average Q-angle of males on both the left and right side was greater for BMI 25-30 kg/m^2^ compared to the BMI less than 25 kg/m^2^ group, and these differences were found to be significant (Figure 7). The average Q-angles of females on both the left and right sides were greater for BMI 25-30 kg/m^2^ compared to the BMI less than 25 kg/m^2^ group, and these differences were found to be significant (Figure 8).

Among males, the right Q-angle and left Q-angle both showed significant positive correlations with height, weight, BMI, right femur length, left femur length, right bicondylar distance, and left bicondylar distance (p<0.05). The highest correlation was found between weight and BMI (Table 1).

**Table 1:** Correlation of Q-angle with various anthropometric variables in males

Correlations (male)	(R)Q-angle p-value	(L)Q-angle p-value
Age	0.758	0.633
Height	0.005	0.008
Weight	0.000	0.000
BMI	0.000	0.000
(R)femur length	0.014	0.020
(L)femur length	0.011	0.017
(R)bicondylar distance	0.012	0.008
(L)bicondylar distance	0.010	0.006

Among females, the right Q-angle showed significant positive correlations with weight and BMI (p<0.05). The highest correlation was found with weight. Left Q-angle showed significant positive correlations with weight, BMI, right femur length, and left femur length (p<0.05). The highest correlation was found with weight (Table 2).

**Table 2:** Correlation of Q-angle with various anthropometric variables in females

Correlations (female)	(R)Q-angle p-value	(L)Q-angle p-value
Age	0.152	0.190
Height	0.103	0.063
Weight	0.000	0.000
BMI	0.000	0.000
(R)femur length	0.055	0.018
(L)femur length	0.053	0.011
(R)bicondylar distance	0.316	0.212
(L)bicondylar distance	0.302	0.226

## Discussion

The quadriceps angle has an important bearing on the biomechanics as well as the overall health of the knee joint [10,20]. It helps in the clinical assessment of the quadriceps muscle pull, thereby majorly affecting the patellofemoral mechanics [7,9]. Thus, any factor or misalignment affecting the Q-angle will affect the normal functioning of the knee joint [3]. This fact is especially important for physically active individuals and athletes, as values of Q-angles can help in predicting knee problems [10]. Since the value of the Q-angle depends upon the position of the ASIS, patella, and tibial tuberosity [7,11,26], a variation in the location of these structures causes the value of the Q-angle to vary, thereby giving a normal range of 8° to 22°, according to various studies. This study was aimed at finding the normal value of the Q-angle and assessing its relation to various anthropometric variants in patients reporting to our setup. Females were found to have greater values of Q-angles, as has been documented in various other studies [4,26], which can be attributed to their wider pelvic diameters as compared to males [4,29], thereby affecting the position of the anatomic points. A laterally placed tibial tuberosity in females contributes to an increase in the Q-angle. A more laterally placed TT in females could be due to an increase in the valgus angle or tibial torsion [28]. Individuals with taller heights are known to have smaller Q-angles [28]. Shorter heights in females as compared to males, along with shorter femur lengths, contribute to higher Q-angle values. In our study, we found that males with higher BMI had higher angle values. The Q-angles were found to be higher on the dominant side in both sexes. We also found that the Q-angle values were related directly to the bicondylar distance.

Thus, given the positive dependence of the Q-angle on various anthropometric parameters, some of which are modifiable, like weight and quadriceps strength, a fair estimation of the quadriceps pull and the possible effects on patello-femoral kinematics can be done. A study using regression modelling will be able to identify modifiable factors independently associated with the Q-angle. The results can possibly have prognostic implications in individuals who are physically active and athletes. Since Q-angle measurement is an inexpensive and easy-to-assess parameter that can be done on an OPD basis, it can be easily used to anticipate, predict, and thus prepare for future problems with adequate physiotherapy.

## Conclusions

The goal of this study was to examine Q-angle values in connection to age, weight, height, gender, dominant side, and intercondylar distance. The Q-angles were found to be greater in females and showed significant positive correlations with weight and BMI. The highest correlation was found with weight. Among males, Q-angles showed significant positive correlations with height, weight, BMI, femur length, and bicondylar distance. The highest correlation was found between weight and BMI. Thus, it can be safely concluded from the study that Q-angles show a positive relation to the weight and height of an individual, with females having higher values, thereby predisposing them to more patellofemoral joint pathologies. Thus, individuals/players with a higher BMI need to realize the possible demerits of strenuous activities and take necessary precautions in the form of focused physiotherapy and reducing their BMI to prevent or decrease the possible damage to the knee joint.
